# A Capacitively-Fed Inverted-F Antenna for Displacement Detection in Structural Health Monitoring

**DOI:** 10.3390/s20185310

**Published:** 2020-09-17

**Authors:** Songtao Xue, Zhiquan Zheng, Shuai Guan, Liyu Xie, Guochun Wan, Chunfeng Wan

**Affiliations:** 1 Department of Disaster Mitigation for Structures, Tongji University, Shanghai 200092, China; xue@tongji.edu.cn (S.X.); 1932510@tongji.edu.cn (Z.Z.); tjguan@tongji.edu.cn (S.G.); 2 Department of Architecture, Tohoku Institute of Technology, Sendai 982-8577, Japan; 3 Department of Electronic Science and Technology, Tongji University, Shanghai 200092, China; wanguochun@tongji.edu.cn; 4 Key Laboratory of concrete and pre-stressed concrete structure of Ministry of Education, Southeast University, Nanjing 210096, China; wan_seu@163.com

**Keywords:** displacement sensor, capacitively-fed inverted-F antenna, resonant frequency

## Abstract

This paper presents a capacitive displacement sensor based on a capacitively fed inverted-F antenna (CFIFA) for displacement detection. The sensor is composed of a grounded L-shape patch and a rectangular upper patch, forming a capacitor between them. The asymmetric dipole model is adopted to explain the frequency shift and current distribution of the proposed antenna sensor at its first-order resonance. The numerical simulation of the CFIFA using the Ansoft high-frequency structure simulator (HFSS) software is carried out to optimize the dimensional parameters, allowing the antenna to perform better. Two sets of CFIFAs are fabricated and tested for verification. Results show that the CFIFA has a good linear relationship between its first resonant frequency and the relative displacement, and is capable of a long range of displacement measuring.

## 1. Introduction

Civil structures form our built environment and affect the human, social, ecological, economic, cultural, and aesthetic aspects of societies [1]. However, in the course of any building’s life, aging, environmental effects, and operational errors can cause severe structural degradation thereby bringing safety and financial concerns [2]. To solve these problems, the health condition of structures needs to be evaluated at periodic intervals, which helps mitigate risks, prevent disasters, and plan maintenance activities in an optimized manner. Structural health monitoring (SHM) emerges as time and circumstance require.

Structural health monitoring (SHM) is a highly active area of research devoted to developing the tools and techniques needed for automatic structural-integrity assessments [3]. For this purpose, sensors are usually deployed to detect structural damage and transmit data to data acquisition systems for signal processing and analysis. Sensors can be divided into wired and wireless types in general. In the SHM field, comprehensive structural monitoring often requires a large-scale deployment of sensors [4]. Wired sensors are expensive to implement for a large-scale application because of labor and wiring costs. Wired sensors also have range limitations based on their power and resolution requirements [5,6].

In recent years, to meet the requirement of large-scale deployment, computer vision-based technologies and wireless sensors have been widely used. Computer vision-based technologies use cameras to capture the origin image, then extract useful features through visual tracking methods and finally obtain the measurands [7]. The technologies have many advantages such as non-contact, long distance, rapid, low cost and labor, and low interference to the daily operation of structures [8]. Nevertheless, since the determination of the measurands is based on the original image, the technologies are susceptible to changes in illumination and weather conditions which affect the quality of the original image the camera gets. Furthermore, the cameras also need power supply for imaging, and while an uninterruptible power supply (UPS) can improve its reliability when the primary power source is lost during emergencies or disasters, it will consume extra expense and add complexity to the system.

As for wireless sensors, they utilize technologize, such as WIFI [9], the general packet radio service (GPRS), ZigBee [10,11], Bluetooth [12] and other telecommunication technologies [13], to realize wireless communication. However, they still need energy from on-board batteries or other methods to function, which will result in reduced reliability, increased installation complexity and cost of wireless sensors, and eventually pose a long-term environmental risk with disposal of billions of batteries [14]. These problems have motivated researchers to develop novel passive wireless sensors to eliminate the power line of wireless sensors.

Passive wireless systems harvest their energy from the environment or collect it from a powered interrogating unit during wireless communication. They have simpler electronics; hence, they can be purchased at lower costs, but their sensing performance is not optimal [5]. Despite the decline in sensing performance, passive wireless sensors continue to prevail because they do not need to be extremely sophisticated or precise while having low cost and acceptable reliability [15].

Generally, passive wireless sensors can be divided into four categories: the surface acoustic wave (SAW)-based sensors [16,17], inductive coupled sensors [18,19], radio-frequency identification (RFID) enabled sensors [20,21,22,23], and chipless antenna sensors [24,25]. Among them, the RFID system, originally used as an electronic device to replace the barcode, has expanded into low-cost, ubiquitous, passive wireless sensing in recent years [26]. In RFID systems, antennas can be periodically activated by interrogation waves and backscattered signals, including information from antenna ID and measurands, which can be received by readers or other antennas. As a result, periodic monitoring for structures can take place at a low cost.

Among the passive RFID-based sensors, patch antenna sensors have been widely adopted in structural health monitoring due to their simple configuration, multimodality, low cost and other advantages [27,28]. Passive RFID-based patch antenna sensors can recognize local damage or a failed bearing capacity of individual members; they also help identify structural deformation in terms of strain or displacement and serve as an important detection measurand in SHM.

Daliri et al. [29] designed a strain sensor based on a circular patch antenna, which shows the linear relationship between strain and resonant frequency shifts. In addition, by applying a meandering technique, Daliri et al. [29] developed a meandered circular microstrip patch sensor, which has demonstrated its omnidirectional strain sensing ability by being threefold more sensitive and five times smaller than a simple circular patch. Yi et al. [30,31] introduced a strain and crack sensor based on a folded rectangular patch antenna integrated with an RFID tag. They pointed out that raising the initial resonant frequency enabled the design of a smaller folded patch antenna and increased its absolute strain sensing sensitivity. Yi et al. [31] also concluded that the change in dielectric constant due to strain affects the resonance frequency shift. Kuhn et al. [32] brought up a novel wireless strain sensor based on the inverted-F antenna (IFA) and focused on its miniaturization and performance optimization.

However, stressed single patch antennas represented by the above antennas are facing the issues of incomplete strain transfer ratio, insufficient strength, etc. [33]. To solve the problems, unstressed patch antenna sensors have been proposed. Xue et al. [34] presented a novel crack sensor based on a rectangular patch antenna with an overlapping sub-patch. The change in overlapped length caused by crack propagation can change the total electric length of the two combined radiation patches and cause resonant frequency changes. However, this kind of antenna sensor is not suitable for long-range displacement detection. Therefore, a long-range displacement sensor based on the capacitively fed inverted-F antenna (CFIFA) was developed by Guan et al. [35]. The feasibility of a CFIFA as a displacement sensor has been proven by numerical simulation, but it has not yet been fabricated for commercial distribution.

Based on the integrated inverted-F antenna [36], where the inverted-F strip is printed on the circuit board, an additional capacitor is introduced between the feeding patch and the L-strip patch. As a result, the feeding patch and L-strip patch can move relatively to each other, which leads to a resonant frequency shift. Their relative movement is correlated with displacement to simplify the antenna design.

This paper presents an improved capacitively-fed inverted-F antenna based on the work of Guan et al. [35]. In this paper, a monopole equivalent model is proposed to explain the variation of the CFIFA’s resonant frequency with displacement. This paper is organized as follows: Section 2 introduces the concept and the sensing principle of the capacitively-fed inverted-F antenna. Section 3 presents the sensor model and numerical simulation results. Section 4 presents the experiment’s instrumentation setup and a comparison of the simulation results with the experimental results. Conclusions are then drawn and future research potential is discussed.

## 2. Design and Theory

This section describes the origin of using the capacitive feed method to design the capacitively fed inverted-F antenna and presents a monopole equivalent model to explain the variation of the CFIFA’s resonant frequency with displacement.

### 2.1. Design of the Capacitively Fed Inverted-F Antenna

To solve the disadvantages of stressed single patch antennas, a capacitor can be introduced into the feed part and the sensing part of the antenna to form a separate structure. This is called the capacitive feed. Vandenbosch and van de Capelle [37] introduced a small patch located between the ground plane and the radiating patch of a microstrip antenna. Fed by a coaxial probe, the small patch can excite the radiating patch through capacitive coupling. Rowell and Murch [38] applied the feed method to a capacitively loaded planer inverted-F antenna and revealed that impedance characteristics can be controlled by varying the dimensions of capacitive feed. Su et al. [39] presented a printed inverted-F monopole antenna using a microstrip-coupled feed and found that adjusting the feeding capacitor obtained good impedance matching of the antennas.

**Figure 1:**
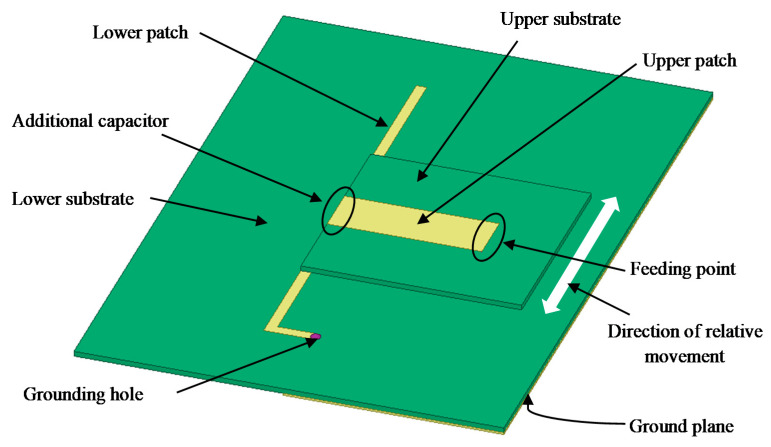
Components of the capacitively-fed inverted-F antenna (CFIFA).

This paper presents the development of a capacitively-fed inverted-F antenna, as shown in Figure 1. It includes two parts: One part consists of a lower substrate designated as the ground plane, a grounding hole, and an L-shaped patch (called the lower patch). Another part consists of the upper substrate and a rectangular upper patch. The overlapping areas of the upper and lower patch and the substrate between them form an additional capacitor. The end of the upper patch is the feeding point. The lower patch is connected to the ground plane via a grounding hole, which helps reduce the antenna size.

**Figure 2:**
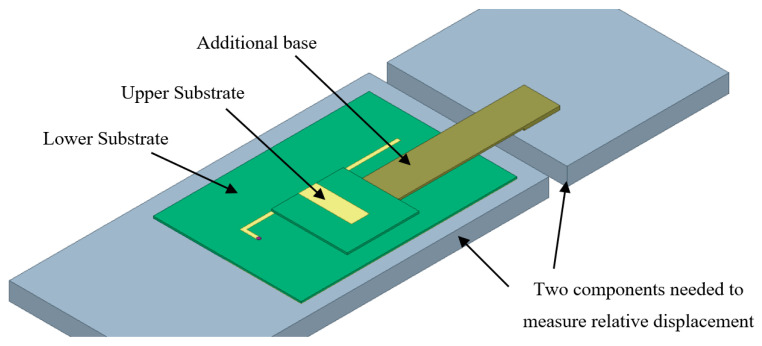
Installation diagram of the CFIFA.

In the application, the upper and lower substrates of the CFIFA are respectively fixed on two components needed to measure relative displacement in structures. To make the antenna easy to install, a suitable additional base is necessary. The CFIFA’s lower substrate is attached to one component, and the upper substrate is attached to the other component through the addition base, as shown in Figure 2. After the sensor’s installation, relative displacement between the two components leads to relative movement along the long side of the L-shaped strip between the upper and lower substrates, which changes the resonant frequency. Since there is no bonding between the upper and lower substrates, they are free to move relatively to each other; therefore, a long-range displacement can be measured.

The proposed sensor is for displacement/deformation with slow change during a long-time service term. Due to the installation characteristics of the sensor, when measuring local displacement, such as expansion joint displacement and crack propagation, the CFIFA-based sensor is convenient for installation and use. In contrast, in terms of global displacement measurement, such as bridges’ deflections and settlement measurement, where the displacement reference is not easily determined, the sensor is difficult for field application.

### 2.2. Theory of the Capacitively-Fed Inverted-F Antenna

The traditional integrated inverted-F antenna is a variant of the monopole. When the monopole is coplanar with the ground plane, the transmission line model can be used to analyze it [40]. In the case of non-coplanar and the ground plane has comparable dimensions to the monopole, the whole integrated inverted-F antenna behaves as an asymmetric dipole, and the ground plane acts as one part of the dipole [36].

**Figure 3:**
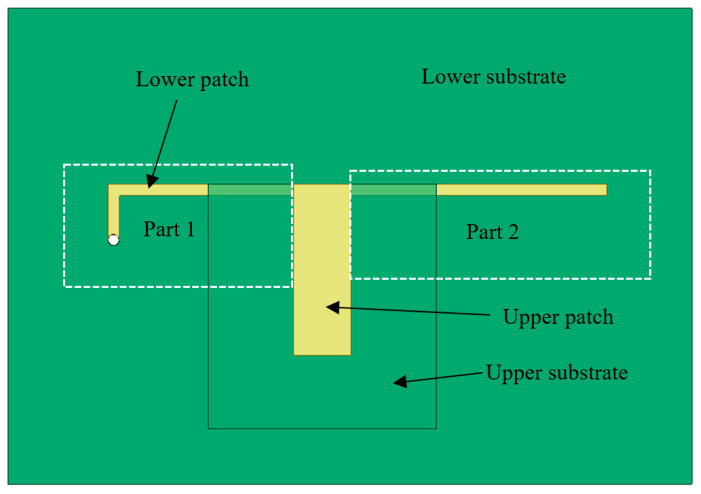
Part 1 and Part 2 of the lower patch.

**Figure 4:**
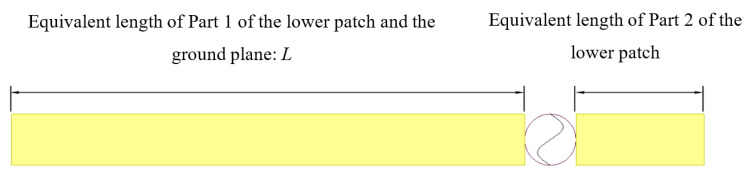
Equivalent asymmetric dipole model of the capacitively fed inverted-F antenna.

When considering the newly developed CFIFA in this paper, the lower patch can also be seen as an asymmetric dipole fed by the upper patch. The additional capacitor divides the L-shape lower patch into two parts as shown in Figure 3: Part 1 is connected to the ground plane via the grounding hole which acts as one part of the asymmetric dipole. Part 2 is on the right side of the upper patch. The ground plane and Part 1 work together to form the domain part that affects the antenna resonance frequency. The equivalent asymmetric dipole model is shown in Figure 4. If Part 2 of the lower patch is ignored, the rest can be regarded as a monopole. The potential resonant frequencies of a printed monopole strip can be estimated by the following formula [41]:

(1) $$f_{n}=a\frac{(2n-1)c}{2\sqrt{\varepsilon_{r}}L},n=1,2,3,\ldots ,$$

where c is the speed of light, L is the length of monopole strip, $\varepsilon_{r}$ is the dielectric constraint of the substrate, and a is a correction factor approximately equal to 0.88.

In terms of our newly developed CFIFA, we assume that the effect of the ground plane is equivalent to a monopole of a certain length. *L* can then be divided into two parts:

(2) $$L_{}=L_{a}+L_{b},$$

where $L_{a}$ represents the length of the equivalent monopole of the ground plane, and $L_{b}$ represents the length of Part 1 of the lower patch.

Verification of the above assumption will be presented in the next section.

## 3. Simulation

**Figure 5:**
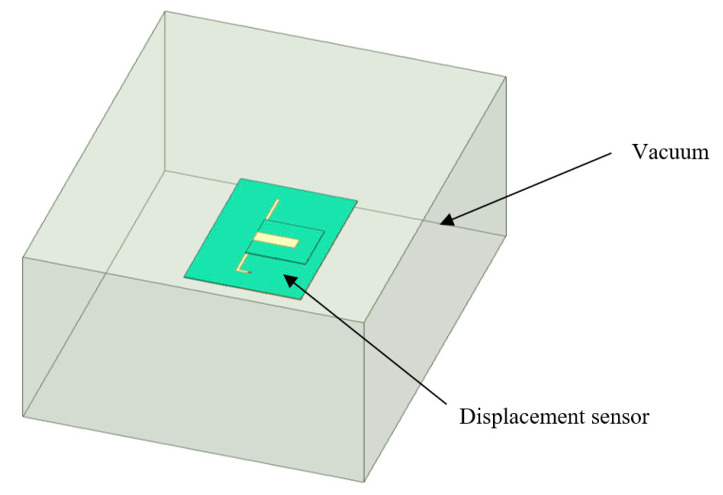
Schematic diagram of the capacitively-fed inverted-F antenna (CFIFA).

The radiation properties of the inverted-F antenna sensor are simulated using the Ansoft high frequency structure simulator (HFSS). The model in the HFSS consists of two parts. One part has a dielectric substrate sandwiched between an L-shaped radiation patch and a ground plane. Another part has a rectangular patch printed on a smaller dielectric substrate, which is shown in Figure 5. The L-shaped radiation patch is connected to the ground plane through a grounding hole. The material of the dielectric substrate is Rogers RT/duroid 5880 (tm) with a dielectric constant 0.22. Copper is chosen as the material for both the radiation patches and the grounding hole. The sensing system is arranged inside an air cuboid with the side’s length of half a wavelength to ensure the computational accuracy of the far-field radiation. The air cuboid is set as radiation boundaries. The antenna is fed by a lumped port connected with the ground plane at the end of the top patch. The ground plane and the patch are set as perfect E boundaries to ensure that the electric field is perfectly perpendicular to the surfaces.

**Figure 6:**
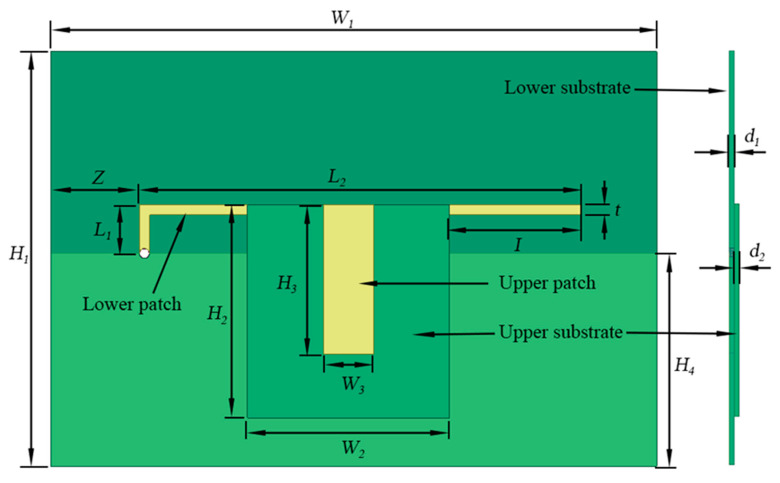
The schematic diagram of each parameter.

After calculating the first order resonant frequency by HFSS, a set of proposed sensor systems with a better performance was selected. The dimension parameters are shown in Table 1, and the schematic diagram of each parameter is shown in Figure 6.

### 3.1. Verification of the Assumption in Section 2

**Figure 7:**
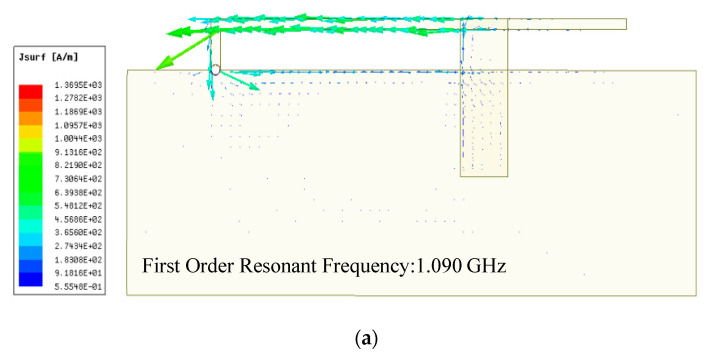

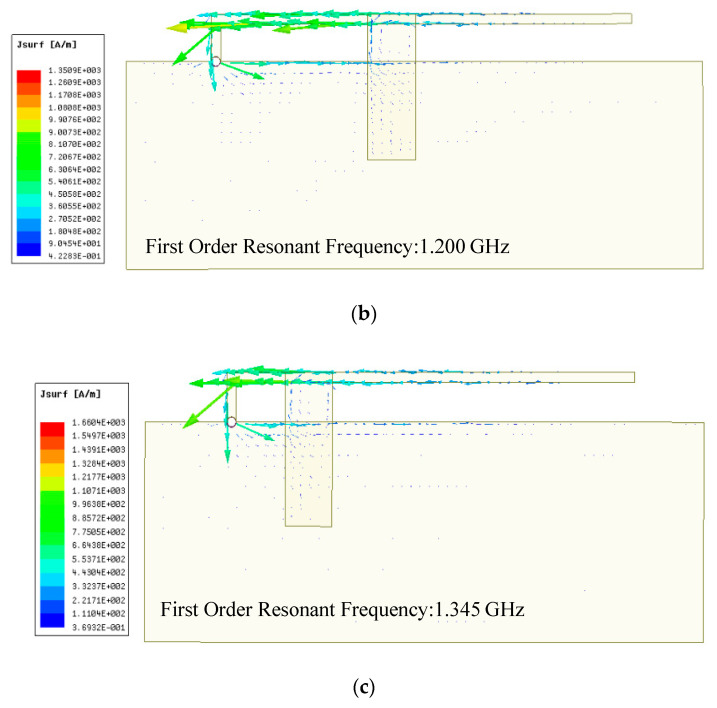
The current distribution at the first order CFIFA resonance. (**a**) *I* = 5 mm, (**b**) *I* = 15 mm, and (**c**) *I* = 25 mm.

The simulation proceeds in a wide range of location variations by HFSS, with the location (represented by symbol *I*) of the upper substrate moving from 0 to 30 mm along the L-shaped radiation patch. When the relative displacement of the upper and lower substrates *I* is 5, 15, 25 mm, the current distribution of the CFIFA at the first order resonant frequency is as shown in Figure 7. At the antenna resonant frequency, the current on the upper surface is mainly distributed in Part 1 of the lower patch and through the grounding hole into the ground plane. This proves that at the first order resonance, the proposed CFIFA behaves as an asymmetric dipole. The ground plane and the Part 1 of the lower patch together constitute the main resonance length of the antenna. With the upper patch moving from right to left, the resonance length decreases, and the resonance frequency increases, which is consistent with Equation (1).

**Figure 8:**
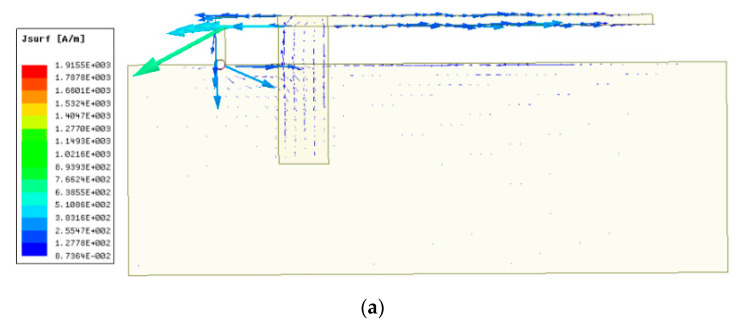

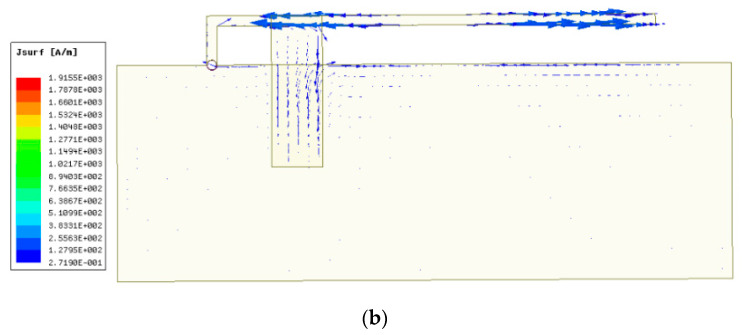
The current distribution of the CFIFA when *I* = 25 mm. (**a**) Second order resonance and (**b**) third order resonance.

To determine whether Equation (1) is applicable to higher CFIFA resonant frequencies, the current distributions at the second and third order resonance are as shown in Figure 8. For a higher resonance, the current in Part 2 of the lower patch cannot be ignored, making Equation (1) no longer applicable. It is necessary to consider the influence of Part 2 of the lower patch to analyze a higher resonance of the CFIFA. 

### 3.2. Performance Simulation

**Figure 9:**
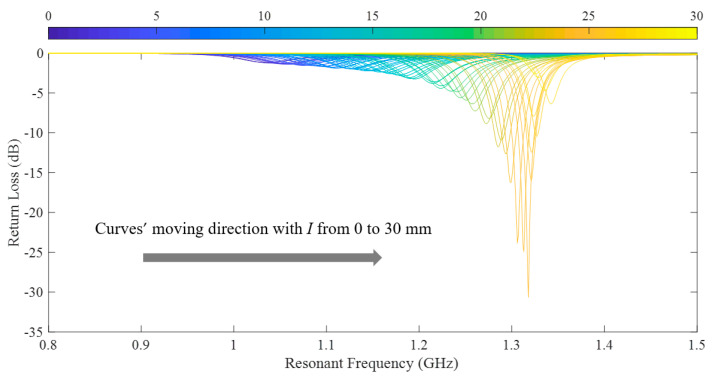
The return loss curves of simulation.

As the upper substrate moves from 0 to 30 mm along the L-shaped lower patch, the return loss curves of the CFIFA around the first order resonant frequency are acquired for each step with 0.5 mm/step, as shown in Figure 9.

**Figure 10:**
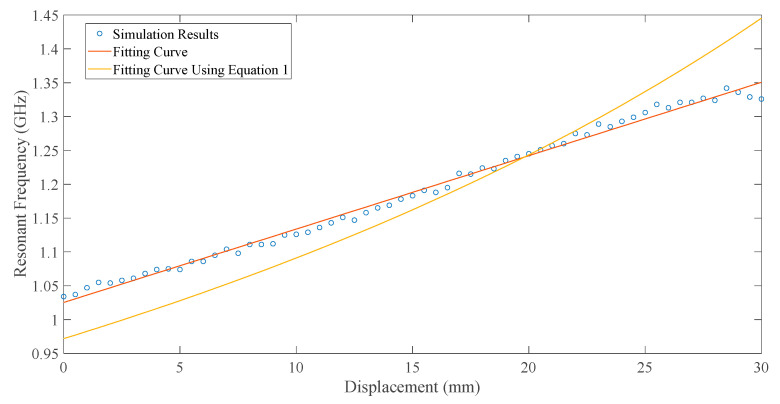
The relationship between resonant frequency and displacement.

The first order resonant frequency of the capacitively-fed inverted-F antenna is extracted from the return loss curve at each moving step. The scatter diagram of the resonant frequency and displacement of the upper substrate is plotted in Figure 10. By fitting the simulation results linearly, the obtained sensitivity of the inverted-F antenna is 10.8 MHz/mm, and the correlation coefficient is 0.9966. 

According to the assumption in Section 2, the effect of the ground plane can be equivalent to a monopole of a certain length $L_{a}$. The $L_{a}$ obtained by data fitting is 55.47 mm, and the curve is expressed in Equation (1) and shown in Figure 10. In the case of the relative displacement of upper and lower substrates greater than 20 mm, with an increase in the relative displacement, the fitting curve of Equation (1) gradually deviates from the simulation result. Therefore, as Part 2 of the lower patch gets longer, its effect cannot be ignored.

According to the simulation results, the displacement of the upper substrate of the antenna has a good linear correlation with the resonant frequency. However, many differences can occur between numerical simulation and practical application. These differences can be caused by the effects of radiation, surrounding environment, boundary condition, etc. To pinpoint the causes and their effects, more practical experiments are still necessary.

## 4. Experiment

For this experiment, we made two sets of the inverted-F antenna with the same dimensions as designed in the simulation stage. Copper was selected as the material of all printed patches and the grounding hole, and RT5880 was used for making the substrates.

### 4.1. Instrumentation Setup

**Figure 11:**
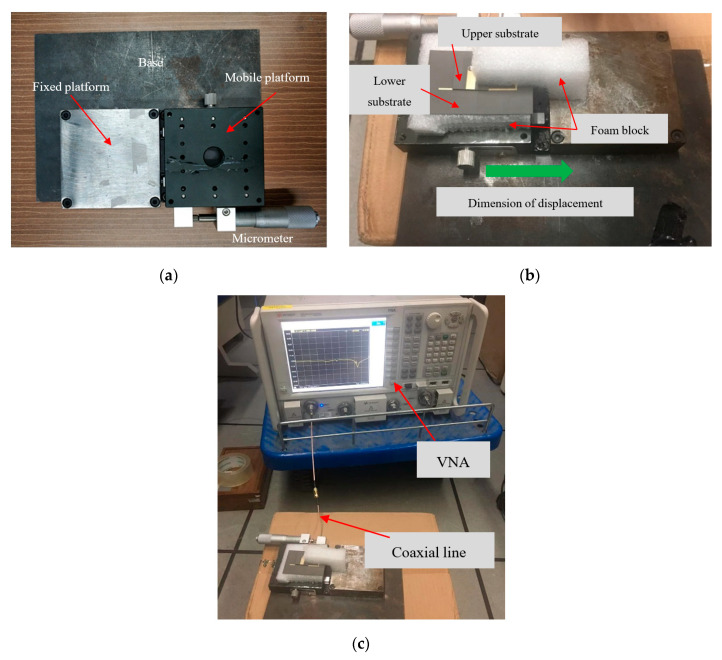
The experimental setup. (**a**) The crack gauge, (**b**) the CFIFA on the crack gauge, and (**c**) the CFIFA connected to the vector network analyzer.

To verify the simulation results, we tested two groups of antennas with the same parameters. To ensure the accuracy of the relative displacement between upper and lower substrates, the testing sensing system was established as shown in Figure 11. The upper and lower substrates of the inverted-F antenna are respectively fixed to two metal blocks of the crack gauge. In order to reduce the influence of the metal block on the antenna radiation, a foam block is used to separate the lower substrate of the antenna from one metal block of the crack gauge; meanwhile, the upper substrate is connected to another metal block through another foam block. The relative displacement between the two metal blocks of the crack gauge is also the relative displacement between the upper and lower substrates of the antenna. The relative displacement between the two metal blocks of the crack gauge is controlled by a micrometer, which can achieve high accuracy. The inverted-F antenna connects to a vector network analyzer (VNA) via a coaxial line with one end tin-soldered to the feeding point of the antenna and another end connected to the vector network analyzer through a tin-soldered Sub-Miniature-A (SMA) connector.

**Figure 12:**
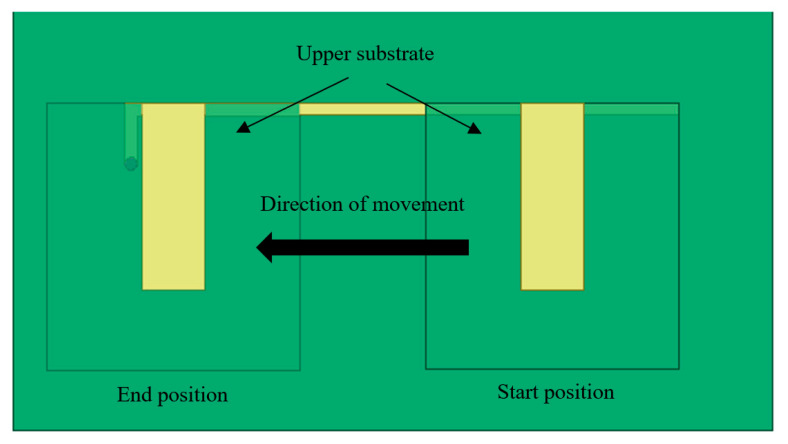
The starting and ending positions of the upper substrate.

When the test started, the upper substrate of the CFIFA was located at one end of the lower patch away from the grounding hole (*I* = 0 mm). Then, by adjusting the micrometer of the crack gauge, the upper substrate was controlled to move along the lower patch toward the grounding hole with 0.1 mm incremental steps. The VNA sent a sweeping signal from 1 to 2 GHz to the antenna and monitored the backscattering signal from the antenna to obtain the return loss curve of the sensor for each step. For reliability of data, each incremental step stayed for 30 s and then recorded the data after the return loss curve of VNA becoming stable. The test ended when the upper substrate reached the end of the lower patch with the grounding hole (*I* = 30 mm). The starting and ending positions of the upper substrate are shown in the Figure 12.

After the test, we obtained the shift of the resonant frequency by extracting the resonant frequency at the local minimum of each return loss curve.

### 4.2. Experimental Results

**Figure 13:**
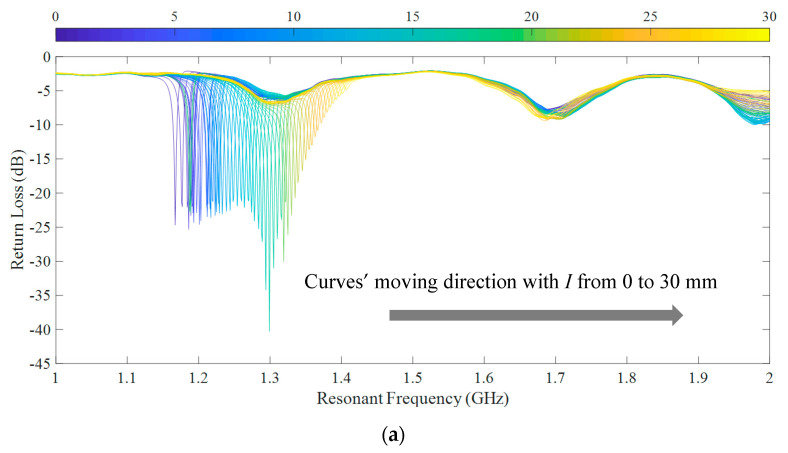

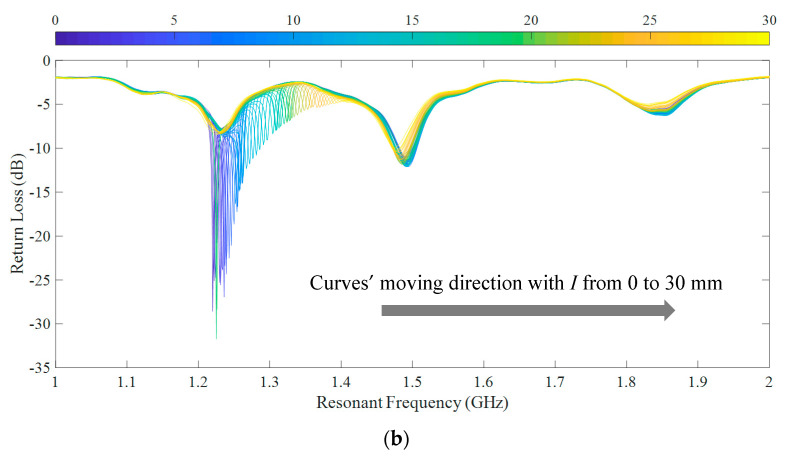
The return loss curves of experiment. (**a**) Group 1 and (**b**) Group 2.

Return loss curves of the two groups of CFIFAs are shown in Figure 13. The abscissa corresponding to the first local minimum point of each curve in the figure is the first-order resonant frequency of the CFIFA. The other two local minimum points of the curve are caused by environmental influence rather than the resonant point of the CFIFA, because the frequency of the two groups of CFIFAs at the two local minimum points varies considerably.

By comparing the return loss curves of the two groups of CFIFAs, we find that group 1 has better impendence matching at the first resonant frequency than group 2. This may be caused by the difference in welding between the two groups’ connections between the coaxial line and the feed point of the upper patch.

**Figure 14:**
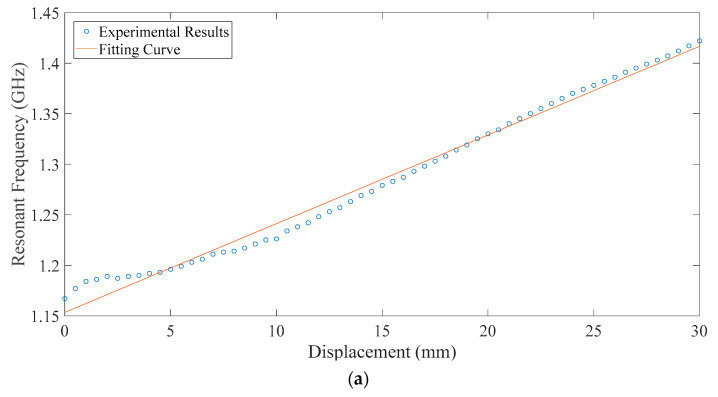

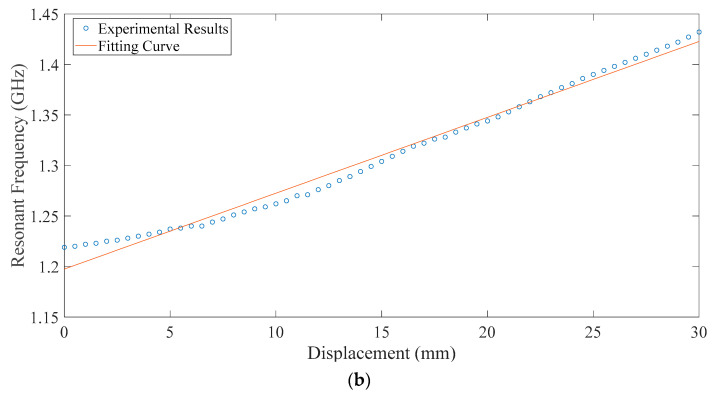
The resonant frequency with respect to displacement. (**a**) Group 1 and (**b**) Group 2.

For each incremental step, the resonant frequency can be extracted from the corresponding return loss curve recorded in the test. The scatter diagram of the resonant frequency and relative displacement between the upper and lower substrates for each group are plotted in Figure 14. The fitting curve is obtained by linear fitting with MATLAB. Then, the sensitivity and the correlation coefficient of each set of antennas are obtained. The experimental results also show a good linear relationship between the displacement and resonant frequency of the CFIFA.

## 5. Results and Discussion

Based on the simulation results and the experimental results of the CFIFA, the sensitivity and correlation coefficient of the fitted line are given in Table 2. Additionally, the performance parameters of some other passive wireless displacement sensors are listed in Table 3.

According to Table 2, for the two groups of CFIFAs in experiment, the correlation coefficients of fitting curves are very similar, but the sensitivity varies considerably. This is due to the deviation of the two groups of CFIFAs in the wielding, fabrication and test environment.

For simulation results and experiment results, both results indicate a great linear relationship between the relative displacement of the lower and upper substrates of the inverted-F antenna (which represents the displacement of structure) and the resonant frequency. However, the correlation coefficient of the fitted lines and the sensitivity of both displacement sensors in the experiment is worse than the numerical results, which is probably due to the following reasons:

(1) The welding of the feeding point and environmental interference may cause some errors, which are not considered in the simulation.
(2) In the practical experiment, it is impossible for the upper and lower substrates of the inverted-F antenna to sit close enough to each other to achieve an air-tight connection. This results in an air film between the two substrates, and this is not considered in the simulation.
(3) Antenna machining errors. While the antenna fabrication is batch processing, the substrate or patch is not completely flat, and the dimensions of each part of the antenna are also subject to some errors.
(4) The upper substrate of the antenna produces translation and rotation away from the direction of the relative displacement. In the simulation of the antenna sensor, the upper substrate moves along the lower patch. However, in the experiment, due to manual operation, it is inevitable to produce a tiny translation or rotation out of the relative displacement direction, which can affect the electromagnetic characteristics of the antenna.

There are a number of displacement sensors in Table 3. The helical antenna [27] is for built-in application; the patch antenna fed by capacitive microstrip lines [33] and the patch antenna with overlapping sub-patch [34] have great sensitivity with small measuring range, which is suitable for crack propagation monitoring; the sensor with complementary split-ring resonators [44] can measure two-dimensional displacement; and the inverted-F antenna-based sensor [45] is designed for the measurement of displacement on metal surfaces. Compared with the sensor in [23], the CFIFA-based sensor has higher sensitivity, and by adjusting the dimensions of the CFIFA, the CFIFA-based sensor can obtain a larger measuring range. Compared with the sensor in [43], the CFIFA-based sensor has a greater measuring range and a similar sensitivity. For conditions where a large measuring range is required and high sensitivity is not required, the CFIFA is suitable.

For practical use, wireless interrogation is suggested to retrieve information for the displacement sensor. An RFID chip is integrated with the inverted-F antenna, and the resonant frequency of the sensing system can be identified by finding the active frequency with the minimum interrogation energy. Data processing methods are also needed to reduce the errors caused by environmental interference.

## 6. Conclusions

In this paper, a capacitively-fed inverted-F antenna for displacement detection was introduced. The equivalent monopole model was used to explain its frequency shift and current distribution at first order resonance. The feasibility of this method was proven by the results of numerical simulation. Then, two CFIFA models with the optimum size obtained by the simulation were fabricated and tested in a wired-testing environment. The experimental results are in agreement with the simulation results and consistent with the theoretical analysis. According to the experiment results, the sensitivity of the CFIFA is 8.15 MHz/mm on average within an effective measuring range of 30 mm.

## Figures and Tables

**Table 1:** Dimension parameters of the chosen group (Unit: mm).

*H*_1_	*W*_1_	*H*_2_	*W*_2_	*H*_3_	*W*_3_	*H*_4_	*L*_1_	*L*_2_	*Z*	*t*	*d*_1_	*d*_2_
41.1	60	21.1	20	14.8	5	21.1	4.8	43.8	8.8	1	0.508	0.508

Note: *H*_4_ is the height of the ground plane.

**Table 2:** Experiment results and simulation results.

Case	Group	Sensitivity (MHz/mm)	Measuring Range (mm)	Correlation Coefficient
Simulation	/	10.8	30	0.9966
Experiment	Group1	8.8	30	0.9938
	Group2	7.5	30	0.9931

**Table 3:** Performance parameters of some passive wireless displacement sensors.

Sensor type	Sensitivity (MHz/mm)	Measuring Range (mm)	Application Scenarios	Reference
Displacement meters based on chipped circular patch antenna	0.28	270	Long-range displacement	[23]
Helical antenna	0.616	7	Built-in	[27]
Patch antenna fed by capacitive microstrip lines	146.8	1	Crack propagation	[33]
Patch antenna with overlapping sub-patch	120.24	1.5	Crack propagation	[34]
Liquid antenna	31.25	4	Liquid surface displacement	[42]
Metamaterial-based sensor	4.48~12.7	20	Displacement	[43]
Sensor with complementary split-ring resonators	50~110	4	Two-dimensional displacement	[44]
Deformation sensor based on an inverted-F antenna	0.7	0.2	Metal surface displacement	[45]
